# CAR T-cell therapy and critical care: A survival guide for medical emergency teams

**DOI:** 10.1007/s00508-021-01948-2

**Published:** 2021-10-06

**Authors:** Anna S. Messmer, Yok-Ai Que, Christoph Schankin, Yara Banz, Ulrike Bacher, Urban Novak, Thomas Pabst

**Affiliations:** 1 Department of Intensive Care Medicine, Inselspital, Bern University Hospital, University of Bern, Freiburgstrasse 10, 3010 Bern, Switzerland; 2 Department of Medical Oncology, Inselspital, Bern University Hospital, University of Bern, Bern, Switzerland; 3 Department of Neurology, Inselspital, Bern University Hospital, University of Bern, Bern, Switzerland; 4 Institute of Pathology, University of Bern, Bern, Switzerland; 5 Department of Haematology and Central Haematology Laboratory, Inselspital, Bern University Hospital, University of Bern, Bern, Switzerland

**Keywords:** Lymphoma, Chimeric antigen receptor T‑cells, Cytokine release syndrome, Immune effector cell associated neurotoxicity, Medical emergency team

## Abstract

Chimeric antigen receptor (CAR) T‑cells are genetically engineered to give T‑cells the ability to attack specific cancer cells, and to improve outcome of patients with refractory/relapsed aggressive B‑cell malignancies. To date, several CAR T‑cell products are approved and additional products with similar indication or extended to other malignancies are currently being evaluated. Side effects of CAR T‑cell treatment are potentially severe or even life-threatening immune-related toxicities, specifically cytokine release syndrome (CRS) and immune effector cell-associated neurotoxicity syndrome (ICANS). Consequently, medical emergency teams (MET) are increasingly involved in the assessment and management of CAR T‑cell recipients. This article describes the principles of CAR T‑cell therapy and summarizes the main complications and subsequent therapeutic interventions aiming to provide a survival guide for METs with a proposed management algorithm.

## Background

The armamentarium of cancer therapies has rapidly evolved within the past two decades. Among recent approaches, targeting the patient’s immunologic self-defence and enhancing T‑cell responses represent very promising developments. Checkpoint inhibitors, chimeric antigen receptor (CAR) T‑cells, and bispecific antibodies attack cancer cells by activating immune effectors and/or decreasing their immune tolerance making them more vulnerable to the effects of immune system cells [1].

To date, several CAR T‑cell products, e.g. tisagenlecleucel (Kymriah®), axicabtagene ciloleucel (Yescarta®), brexucabtagene autoleucel (Tecartus®), and lisocabtagene maraleucel (Breyanzi®) are on the market, although some of them not yet approved by the European Medicines Agency (EMA). Most of them are targeting the CD19 antigen, expressed by most B‑cell malignancies [2–6]. In patients with relapsed or refractory (r/r) large B‑cell lymphoma and patients with mantle cell lymphoma, CAR T‑cell therapies have been shown to induce a temporary or even durable complete remission in the majority of patients [4, 5, 7]. In patients with relapsed B‑ALL following allogeneic hematopoietic stem cell transplantation, CAR‑T cell therapy provides the only curative approach [6]. In March this year, the first CAR‑T cell product against multiple myeloma (idecabtagene vicleucel) has been approved, targeting the B cell maturation antigen (BCMA) [8]. In the coming months and years, approvals for additional CAR‑T cell products for these indications and other B‑cell malignancies are expected.

After CAR‑T cell administration, patients can develop specific acute toxicities, namely cytokine release syndrome (CRS) [9–12] or immune effector cell-associated neurotoxicity syndrome (ICANS) [10, 12], that can vary from minor transient symptoms up to life-threatening conditions. Therefore, early recognition by the oncologic team with timely involvement of critical care teams, i.e. medical emergency teams (MET), is crucial. This review article is a survival guide for METs with a summary of the key toxicities associated with CAR‑T cell therapy and a proposal for a management algorithm.

## Principles of CAR T‑cell therapy

Autologous CAR‑T cells are “living drugs” and are tailor-made for each individual patient. Therefore, a variety of logistic challenges and a complex production process precede CAR‑T cell treatment [13]. In many instances, bridging strategies including cytotoxic compounds, B‑cell antibodies, or radiotherapy, are needed to control the B‑cell malignancy before the patient enters CAR‑T cell therapy. Prior to CAR‑T cell infusion, patients receive lymphocyte-depleting chemotherapy, to ensure tolerance and persistence of the administered CAR‑T product [2]. In the weeks (and sometimes months) following CAR‑T cell infusion, both CD19 and BCMA directed CAR‑T cells show a wide range of interindividual expansion and proliferation dynamics in the recipient and subsequently exert their effects towards the malignant B‑cell tissues as well as the healthy B‑cell compartment.

## Key toxicities related to CAR T-therapy

The American Society for Transplantation and Cellular Therapy (ASTCT) developed a recommendation for definition and grading for CAR T-related toxicity (Table 1; [10]). The grading systems are incorporated and easy to calculate on the CARTOX app, which has been developed by the MD Anderson Cancer Center in the USA [14]. The incidence and severity of CAR T-related toxicities are likely associated with high disease burden, a higher peak of CAR‑T cell expansion in the peripheral blood, and high baseline inflammatory activity [11, 15–18]. Side effects of CAR‑T cell therapy seems to be more pronounced in patients with ALL as compared to DLBCL [4, 6]. Studies in patients treated for ALL reported a significant correlation between neurotoxicity and the presence of CRS as well as its severity [18]. In addition, the different CAR‑T cell products have different rates of toxicities: Axicabtagene ciloleucel causes higher rates of ICANS than tisagenlecleucel [3, 4, 19].

**Table 1:** ASTCT CRS and ICANS consensus grading for adults [10]

	Grade 1	Grade 2	Grade 3	Grade 4
*CRS parameter*				
Fever^a^	≥ 38.0	≥ 38.0	≥ 38.0	≥ 38.0
		With		
Hypotension	None	Not requiring vasopressors	Requiring vasopressors +/− vasopressin	Requiring multiple vasopressors (excl. vasopressin)
		And/or		
Hypoxia	None	Requiring low-flow nasal cannula (≤ 6 L/min)	Requiring high-flow nasal cannula (> 6 L/min) facemask, nonrebreather mask, or venturi mask	Requiring positive pressure (e.g. CPAP, BiPAP, and mechanical ventilation)
*Neurotoxicity domain*				
ICE score^b^	7–9	3–6	0–2	0 (patient is unarousable and unable to perform ICE)
Depressed level of consciousness	Awakens spontaneously	Awakens to voice	Awakens only to tactile stimulus	Patient is unarousable or requires vigorous or repetitive tactile stimuli to arouse. Stupor or coma
Seizure	N/A	N/A	Any clinical seizure, focal or generalized, that resolves rapidly or non-convulsive seizures on EEG that resolve with intervention	Life-threatening prolonged seizure (> 5 min); or repetitive clinical or electrical seizures without return to baseline in between
Motor findings	N/A	N/A	N/A	Deep focal motor weakness such as hemiparesis or paraparesis
Elevated ICP/cerebral oedema	N/A	N/A	Focal/local edema on neuroimaging	Diffuse cerebral oedema on neuroimaging; decerebrate or decorticate posturing; or cranial nerve VI palsy; or papilledema; or Cushing’s triad

*ASTCT* American Society for Transplantation and Cellular Therapy, *CRS* cytokine release syndrome, *ICANS* immune effector cell-associated neurotoxicity syndrome, *CPAP* Continuous Positive Airway Pressure Therapy, *BiPAP* Bilevel Positive Airway Pressure Therapy

^a^Fever is the sole symptom required for classification as grade 1. In patients who have CRS who undergo antipyretic or anticytokine therapy (tocilizumab or steroids), fever is no longer required to grade subsequent CRS severity, CRS grading is then defined by hypotension and/or hypoxia

^b^*ICE* immune effector cell-associated encephalopathy score: 1 point for each: year, month, name city, hospital, one point for every object correctly identified (max. 3 points), follow command, write a standard sentence, and count backwards from 100 by 10. Score of 10: no impairment. CRS and ICANS grades are determined by the most severe event not attributable to any other cause

### Cytokine release syndrome (CRS)

The CRS is the most common acute toxicity following CAR‑T cell therapy with a reported incidence between 37% and 93% across different studies. [3, 4, 6, 20, 21]. The onset of CRS typically occurs within hours up to 4–7 days after CAR‑T cell infusion, but late occurrence up to 14 days has also been reported [3, 4, 6, 20, 22].

The CRS is characterized by excessive immune reaction triggered by various factors, such as infections and immune-modulating drugs, in particular T‑cell engaging treatment strategies [17, 23].

Increased IL‑6 levels correlate with the onset of severe CRS symptoms [24] and seem to play a key role in CRS pathophysiology, as they are higher in patients with CRS than in patients without [25–28]. To date, there is no evidence that T‑cells or CAR‑T cells are a significant source of IL‑6 production [29, 30].

The leading symptom is fever, defined as ≥ 38.0 °C, and is required for a diagnosis of CRS [10]. Other nonspecific symptoms, including malaise, myalgia, fatigue, gastrointestinal complaints (nausea, vomiting, diarrhea), tachycardia, and rash, may also be present [9, 16, 17, 31]. The CRS may be self-limiting and can resolve with supportive care alone or may become life-threatening with capillary leak leading to pulmonary edema, hypotension, multiorgan failure, and circulatory collapse [9, 20], see also Table 1.

### Immune effector cell-associated neurotoxicity syndrome (ICANS)

The ICANS was reported to become manifest in around 40% of CAR‑T cell recipients [32]. The onset of neurotoxicity typically occurs after the start of CRS, and sometimes even after CRS has completely resolved [18]. Typically, ICANS becomes clinically manifest within 4–10 days following CAR‑T cell infusion [3, 4, 20].

The mechanisms underlying CAR‑T cell-related neurotoxicity are not yet fully understood but disruption of the brain-blood barrier and cerebral edema via cytokine release by CAR‑T seem to be key features of ICANS [3, 18]. In up to 95% of patients, CAR‑T cells could be detected in the cerebrospinal fluid (CSF); however, the numbers did not correlate with the severity of ICANS as they could also be found in patients without neurological pathology [18, 33].

Patients with ICANS often develop a characteristic sequence of neurologic symptoms [10, 31], with tremor, dysgraphia, mild expressive aphasia, apraxia and impaired attention in the initial phase. Particularly, expressive aphasia evolving over a period of hours to global aphasia has been reported as a specific symptom [18]. This characteristic finding of an awake patient who is mute and unable to follow commands distinguishes ICANS from other causes of encephalopathy [31]. Furthermore, neurotoxicity can proceed to diffuse cerebral edema with subclinical or clinical seizures [10].

For the assessment and grading of CAR-related encephalopathy, the so-called immune effector cell-associated encephalopathy (ICE) score has been developed [10]. The latter score has recently replaced the CARTOX-10, both scores incorporate key elements of the mini-mental state examination and evaluate alterations in speech, orientation, handwriting, and concentration (Table 1; [10]).

### Important differential diagnoses

Due to the similarity of symptoms and the immunosuppressed state of the patient it is crucial to exclude sepsis and treat infections appropriately. In a recent study on outcomes in critically ill CAR T recipients, sepsis was one of the main reasons for ICU admission [34]. Sepsis-related encephalopathy and meningitis/encephalitis can mimic ICANS. Moreover, severe CRS has been associated with a higher infection risk [35]. The reason for this association is unclear: high cytokine levels, immunosuppressive therapies, aggressive supportive care, and intensive care unit (ICU) management might play a role. Tumor lysis syndrome can usually be distinguished by typical laboratory findings, such as hyperuricemia, hyperkalemia, hypocalcemia, and hyperphosphatemia [36]; however, tumor lysis syndrome and CRS may occur coincidentally [37]. Furthermore, the progression of the underlying malignancy may cause tumor-associated fever and other clinical, metabolic, and imaging abnormalities that resemble those of CRS. CRS is reminiscent of macrophage activation syndrome (MAS) as well as hemophagocytic lymphohistiocytosis (HLH). Both represent syndromes with dysregulated immune response resulting in a severe cytokine storm, hence laboratory findings and the cytokine profile are closely related between MAS/HLH and severe CRS [38]. Thus, MAS/HLH might be regarded as consequence of CRS and CAR‑T cells acting as a trigger for subsequent development of HLH/MAS [39]. In addition, fungal infections as well as fulminant relapses of the underlying disease might be triggers for HLH/MAS [40], which is why consistent and repeated investigation are important once a diagnosis of HLH following CAR‑T therapy has been established.

Hypersensitivity reactions may appear after CAR T infusions and typically present with rash and urticaria, fever, dyspnea, hypotension, and gastrointestinal symptoms and eventually cardiorespiratory failure. Considering ICANS, important differential diagnoses are intracranial bleeding (especially when thrombocytopenia is present) or stroke. ICANS can result in epileptic seizures, which have to be identified in an electroencephalogram (EEG), especially when nonconvulsive.

## Management of CAR T-cell-related toxicities

**Fig. 1:**
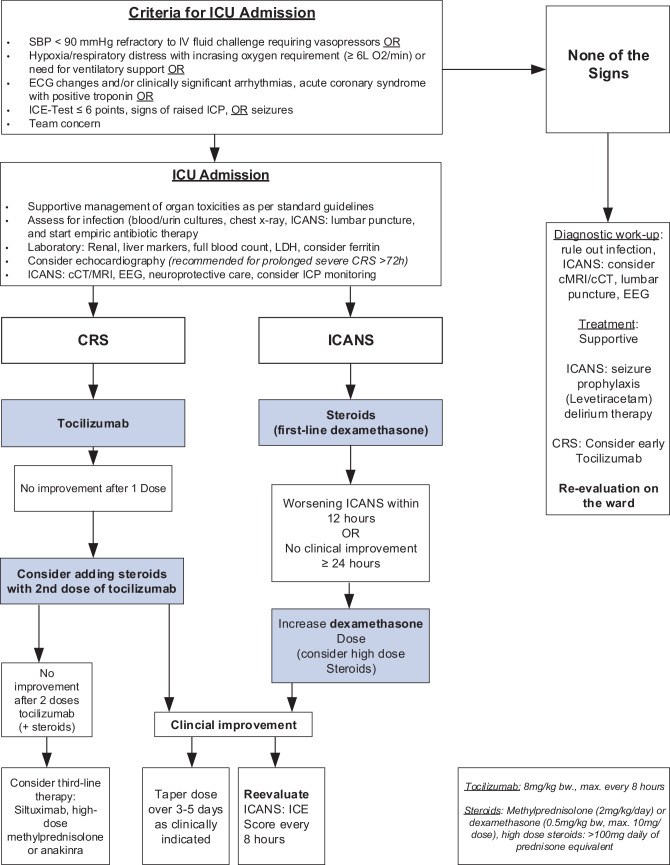
Medical emergency team (MET) algorithm for the assessment and management of CAR‑T related toxicities. Algorithm adapted from Lee et al. [10], Park et al. [12], and Maus et al. [21]. *CRS* cytokine release syndrome, *ICANS* immune effector cell-associated neurotoxicity syndrome, *ICE* immune effector cell-associated encephalopathy, *cCT* cerebral computed tomography, *MRI* magnetic resonance imaging, *ICP* intracranial pressure, *LDH* lactate dehydrogenase, *EEG* electroencephalogram

Timely recognition and interdisciplinary management are key components for the management of patients developing CAR T-related toxicities. The CARTOX group recommends a grade-based management approach for CRS and neurologic toxicity [9, 10, 15]. According to these guidelines and management recommendations, lower grade ICANS and CRS can be managed on the ward with supportive and/or pharmacologic therapy. Management of early stages of CRS and ICANS (grade 1–2) includes ruling out infections and other important differential diagnoses. Whilst most of the patients respond well to supportive care including antipyretics and intravenous fluid hydration, early administration of tocilizumab in patients with lower grade CRS may be increasingly considered standard of care. With respect to ICANS, swallowing assessment and aspiration precautions are recommended. Additionally, cerebral imaging (preferably MRI [21]) and EEG should be considered [41]. In the absence of seizures, prophylactic levetiracetam is recommended [9, 21, 42], but the duration and dose have not yet been determined. In general, an early strong collaboration between MET, hemato-oncologists, neurologists and other organ specialists is crucial. Therefore, at our centr we adapted current CAR T-related toxicity guidelines and developed a concise algorithm to be used by our MET (Fig. 1).

### Criteria for ICU admission

In general, patients with higher (≥ 3) grade of toxicity (see also Table 1) should be managed on the ICU [9, 10, 15, 16, 43]. Thus, patients requiring one or more organ support or have a reduced level of consciousness fulfil the criteria for ICU admission [10, 16]; however, a recently published survey evaluating ICU management of CAR‑T cell-associated toxicities revealed that a majority of ICU admitted patients presented with CRS grade 1–2 (73%) and ICANS grade 2 (81%) [44]. In this survey, reasons for admitting lower grade toxicities were concerns for later deterioration, need for further interventions, concerns for evolving noncardiogenic pulmonary edema, or risk for rapid deterioration due to large tumor burden [10]. The CARTTAS trial investigating outcomes in patients treated with CAR‑T cells revealed that about 30% of the patients required admission to ICU, all for CRS, ICANS, or sepsis [34].

### Intensive care management

Supportive management of organ dysfunction or failure follows respective standard intensive care guidelines [11, 31]. The CAR-ICU survey has shown that management practices are very similar amongst participating units [44]. For the fluid management, most units use repetitive fluid bolus of 4 ml/kg BW, and fluid responsiveness was assessed with noninvasive modes (stroke volume variation, cardiac output, ultrasound guided). Early experience has shown that treating persistent hypotension in CRS with overt fluid management was inferior to early vasopressors use [10]. First line vasopressor in most units is noradrenaline, followed by vasopressin and epinephrine [44]. For patients with respiratory failure, most units perform a noninvasive ventilation trial before intubation [44]. In patients with prolonged severe CRS (persisting for > 72 h without response to intervention), a cardiac assessment, including cardiac biomarkers (e.g. troponin, NT-proBNP/BNP) or the performance of a echocardiography, is recommended [21]. For patients with neurotoxicity, along with neuroprotective measurements, more invasive intracranial pressure monitoring and enhanced neuroprotective treatment (hypertonic saline, mannitol, pharmacological coma) might be necessary [9, 15, 45, 46]. Nonconvulsive and convulsive status epilepticus should be managed with benzodiazepines and additional antiepileptic drugs, preferably levetiracetam, followed by phenobarbital [9]. As sepsis at ICU admission might be an important determinant of mortality in this patient population, particularly when they are neutropenic, screening for infections and commencing broad-band antibiotics has to be considered [34].

### Specific therapies

Corticosteroids are immunosuppressive and are effective in the management of CRS and ICANS [11, 16, 47]. Intravenous corticosteroids are the first-line therapy for patients with ICANS ≥ grade 2 [18], while in CRS they are used for second-line in case of refractory symptoms. Recent recommendations even suggested to add steroid in cases of severe CRS not responding to first dose of tocilizumab [21]; however, the evidence for this is not sound and therefore needs to be considered individually In both CRS and ICANS, methylprednisolone (2 mg/kg and day) or dexamethasone (0.5 mg/kg, maximum 10 mg) are commonly used steroids. To avoid jeopardizing CAR‑T cell function, the initial recommendation of steroid use was limited to patients with CRS refractory to anti-IL‑6 therapy or patients with grade 3–4 CRS toxicities [9]. Today, however, this concern turns out to be unjustified [46, 48–50]. Once CRS symptoms are improving, steroids should be gradually tapered off [21].

Tocilizumab, an IL-6R antagonist, represents the first-line immunosuppressive therapy of CRS [16]. It binds both the soluble and the cell-associated IL‑6 receptor. In several studies, tocilizumab has proven to be effective for severe or life-threatening CRS, and usually patients with CRS rapidly respond to tocilizumab—fever and hypotension often resolve within a few hours [51, 52]; however, considerations have been given to use tocilizumab pre-emptively or even as prophylaxis, as blocking IL‑6 with tocilizumab neither significantly compromised therapeutic efficacy of CAR‑T cells, nor did it negatively affect prognosis of the CAR‑T cell recipients [3, 9, 48, 53]. For patients with isolated ICANS, tocilizumab was shown to be ineffective and should only be administered in patients with concurrent CRS [18, 30, 32]. This might be due to the fact that tocilizumab does not cross the blood-brain barrier [16]; however, IL‑6 is likely involved in the pathogenesis of CAR T-related neurotoxicity and levels of IL‑6 have shown to rise transiently following tocilizumab administration, which might lead to an aggravation of neurologic symptoms [16].

Siltuximab is a direct IL‑6 antagonist and has similar effects as tocilizumab. Siltuximab might have a favorable outcome in the case of passive diffusion of IL‑6 into the CNS [9]; however, a randomized prospective comparison between siltuximab and tocilizumab is still missing [9] and siltuximab has not yet been approved for this indication by US and European drug agencies (FDA and EMA).

For therapy refractory CRS, the addition of third line therapies has been suggested [21]; however, these therapies are currently considered investigational. Whereas IL‑6 blockade prevents mainly CRS, the additional IL‑1 blockade achieved with the use of anakinra could potentially prevent both CRS and ICANS [30]. A recent report suggested that anakinra could be a potential steroid-sparing strategy for the treatment of CAR T‑cell therapy-associated toxicities, mainly ICANS. Several clinical trials investigating its early and/or prophylactic use are ongoing [54].

## Conclusion

In parallel with the introduction of potentially curative CAR‑T cell therapies, the spectrum of unique toxicities is growing as well. Tocilizumab is the mainstay pharmacologic therapy for CRS, while corticosteroids should be reserved for neurologic toxicities and CRS not responsive to tocilizumab. An established multidisciplinary collaboration between dedicated hemato-oncologists, organ specialists and critical care physicians is crucial in the management of these patients. Improved knowledge of CAR‑T related toxicities, and development of new pharmacological options, especially for prevention and therapy of ICANS, will hopefully further increase safety and practicability of CAR‑T cell application in the near future.
